# Dynamic regulation of N^6^,2′-O-dimethyladenosine (m^6^Am) in obesity

**DOI:** 10.1038/s41467-021-27421-2

**Published:** 2021-12-10

**Authors:** Moshe Shay Ben-Haim, Yishay Pinto, Sharon Moshitch-Moshkovitz, Vera Hershkovitz, Nitzan Kol, Tammy Diamant-Levi, Michal Schnaider Beeri, Ninette Amariglio, Haim Y. Cohen, Gideon Rechavi

**Affiliations:** 1 Cancer Research Center, Chaim Sheba Medical Center, Tel Hashomer, Israel; 2 Sackler School of Medicine, Tel Aviv University, Tel Aviv, Israel; 3 The Wohl Institute for Translational Medicine, Chaim Sheba Medical Center, Tel Hashomer, Israel; 4 Department of Psychology, The Hebrew University of Jerusalem, Jerusalem, Israel; 5 The Mina and Everard Goodman Faculty of Life Sciences, Bar-Ilan University, Ramat-Gan, Israel; 6 The Joseph Sagol Neuroscience Center, Chaim Sheba Medical Center, Tel Hashomer, Israel; 7 Mount Sinai School of Medicine, New York, NY USA

**Keywords:** Metabolic disorders, Epigenetics, RNA modification

## Abstract

The prevalent m^6^Am mRNA cap modification was recently identified as a valid target for removal by the human obesity gene *FTO* along with the previously established m^6^A mRNA modification. However, the deposition and dynamics of m^6^Am in regulating obesity are unknown. Here, we investigate the liver m^6^A/m methylomes in mice fed on a high fat Western-diet and in ob/ob mice. We find that FTO levels are elevated in fat mice, and that genes which lost m^6^Am marking under obesity are overly downregulated, including the two fatty-acid-binding proteins FABP2, and FABP5. Furthermore, the cellular perturbation of *FTO* correspondingly affect protein levels of its targets. Notably, generally m^6^Am- but not m^6^A-methylated genes, are found to be highly enriched in metabolic processes. Finally, we deplete all m^6^A background via *Mettl3* knockout, and unequivocally uncover the association of m^6^Am methylation with increased mRNA stability, translation efficiency, and higher protein expression. Together, these results strongly implicate a dynamic role for m^6^Am in obesity-related translation regulation.

## Abstract (web summary)

m6A and m6Am are two prevalent mRNA modifications which are target for removal by the fat mass and obesity gene FTO. Here the authors capture the differential profile of these two modifications in the liver of obese mice and identify dynamic translation regulation by the m6Am modification.

## Introduction

Obesity and overweight are a major concern given their increasing prevalence and associated health problems. Recently, variants within the *FTO* gene—an mRNA demethylating enzyme—were identified as robustly associated with human obesity^1^. While most attention was given to the m^6^A mRNA modification as the first identified demethylated target of *FTO*^2^, new work has demonstrated that *FTO* can also efficiently remove the mRNA m^6^Am cap modification^3^. However, the deposition and possible dynamic involvement of m^6^Am in obesity regulation and metabolism have not yet been characterized.

Various approaches have been employed to attempt explaining the robust association of *FTO* with obesity. Some have implicated m^6^A methylation in metabolic processes such as adipogenesis through control of mRNA splicing^4^; or dopaminergic functioning^5^; while others have found effects of neighboring genes adjacent to *FTO* as possible metabolic contributors^6,7^. Despite the important progress made, a clear picture for the mechanism underlying this association remains elusive. In particular, it is unclear if and in what way does the newly discovered substrate of *FTO,* m^6^Am, regulates obesity. In order to explore the possible involvement, dynamics, and deposition of the two types of mRNA modifications mediated by *FTO*, we systematically explored the m^6^A and m^6^Am methylomes and their functions in obesity-associated metabolic perturbations in high fat Western diet-fed mice and in ob/ob overfeeding mice. In addition, in order to reliably differentiate m^6^Am from m^6^A (which are detected by the same antibody), we constructed a validated map of m^6^Am genes in a context completely free of m^6^A background via knockout (KO) of its methyltransferase, *Mettl3*. Doing so, we were able to reliably and un-confoundingly resolve the controversy concerning the m^6^Am functions (see a detailed description of this controversy below).

**Fig. 1:**
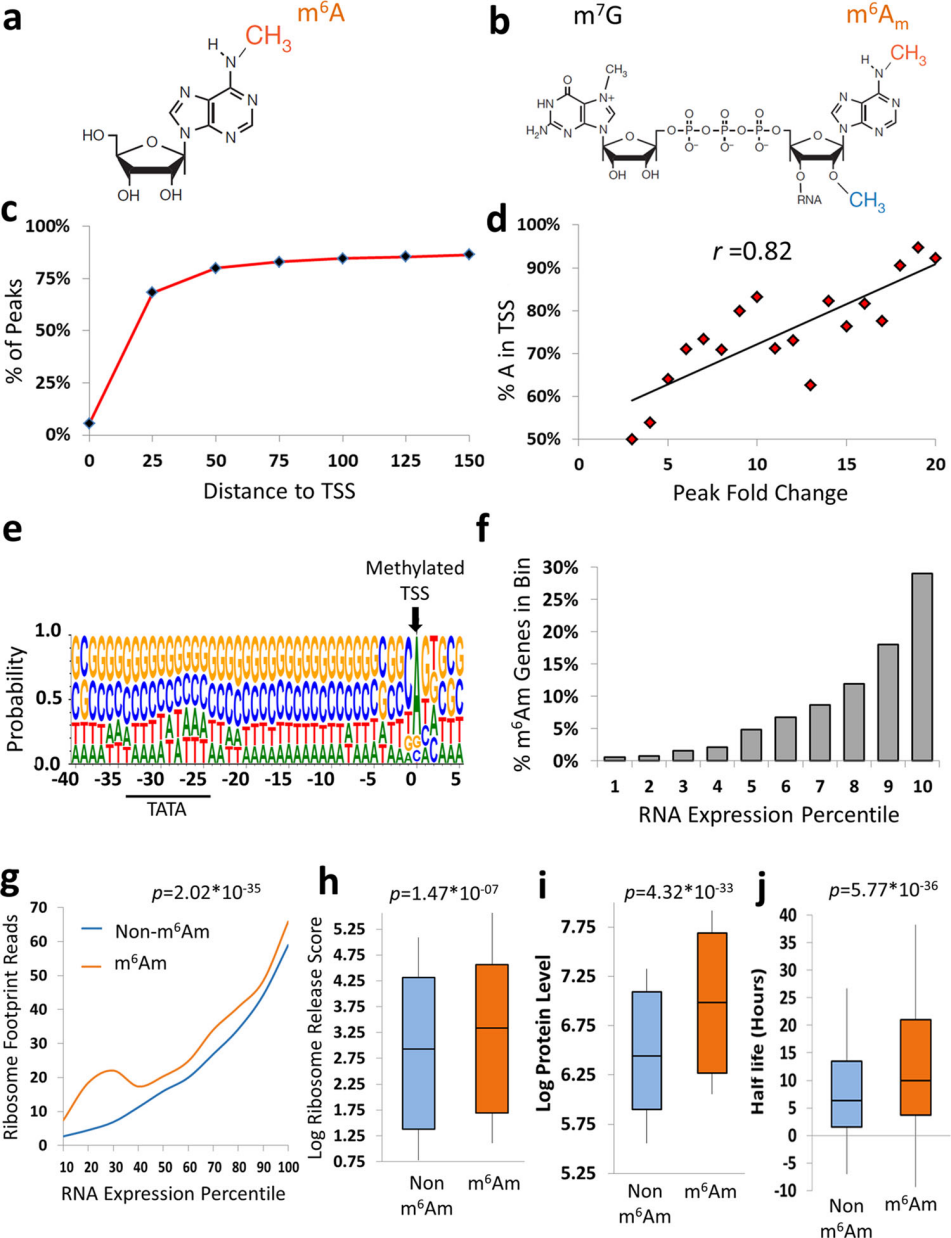
m^6^Am genes are associated with higher translation efficiency, mRNA stability, and increased protein levels in m^6^A depleted mES *Mettl3* KO cells. **a** m^6^A methylation. **b** Cap m^6^Am methylation. **c** Distance of MACS methylation peaks from a reported annotated TSS. **d** Percent of adenosines in annotated TSS as a function of peak fold change. **e** Sequence logo of the m^6^Am peaks around and upstream the annotated TSS within the DNA, portraying the canonical m^6^Am genomic consensus. **f** Fraction of m^6^Am methylated genes across their RNA expression percentile bins. **g** Log ribosome footprint reads of m^6^Am- and non-m^6^Am-decorated genes as a function of their mRNA expression percentile, portraying higher ribosome occupancy of m^6^Am-modified genes above and beyond their expected corresponding RNA level. Two-tailed *p*-value of an ANCOVA analysis controlling for RNA levels of each gene as covariates is reported, F(1,13843) = 155.14, *p* = 2.02 × 10^−35^. **h** Ribosome release scores (RRS) of m^6^Am and non-m^6^Am-modified genes. Two-tailed *p*-value of the Mann Whitney U test is reported, covering a total of 13,240 genes. Box plot surrounds the 1–3 quartiles, whiskers denote 1.5 interquartile range. **i** Log high-throughput proteomic profiling of m^6^Am- and non-m^6^Am-modified genes. Two-tailed *p*-value of the Mann Whitney U test is reported, covering a total of 3970 genes. Box plot surrounds the 1–3 quartiles, whiskers denote 1.5 interquartile range. **j** mRNA half-life measurements of m^6^Am- and non-m^6^Am-modified genes. Two-tailed *p*-value of the Mann Whitney U test is reported, covering a total of 19,649 genes. Box plot surrounds the 1–3 quartiles, whiskers denote 1.5 interquartile range. Source data are provided as a Source Data file.

Though the existence of chemical modifications of RNA molecules has been known for decades, only recently the development of designated antibody-based capturing coupled with next generation sequencing, allowed to map these modifications at high throughput and to begin exploring their physiologic functions systematically (see for example^8,9^; for reviews^10,11^). Mapping the human and mouse m^6^A methylomes deciphered the non-random deposition of this modification alluding to its conserved functions^12,13^. m^6^A is the result of methylation at the N^6^ position of the adenosine (Fig. 1a) in mRNA, generally installed at a RRACH RNA consensus sequence and particularly enriched around stop codons and the 3′ UTR, as well as long internal exons^12,13^. The m^6^Am modification, on the other hand, lays at the first transcribed adenosine, adjacent to the cap 7-methyl Guanosine (m^7^G). It is usually methylated at the 2′-*O* position (Am), and can be further methylated at the *N*^6^ position to form an *N*^6^, 2′-*O*-dimethyladenosine (m^6^Am) (Fig. 1b). mRNA cap-m^6^Am is typically installed in a context of a BCA consensus sequence within the DNA (where B denotes G or C or T, and A is the methylated adenosine^14^). The extent of m^6^Am was reported to be 2–15 times higher than Am in most tissues^15^, making it a rather abundant cap modification.

Both m^6^A and m^6^Am are recognized by anti-m^6^A antibodies used in m^6^A-seq methods^12,16^. This makes it particularly difficult to reliably differentiate between the two modified nucleotides, and especially since m^6^A is also present in the 5′ UTR. Using a single nucleotide resolution approach allowed unequivocal detection of only 24 sites localized exactly on the annotated transcription start site position (TSS) in one study^14^ or 46 in another^3^. This small number of sites detected using this approach is partially due to the difficulty in determining the specific site where transcription begins given the vast variability in promoter regions and transcription initiation^17–19^. Finding a way to identify multiple m^6^Am peaks reliably is critical in order to confidently and reliably decipher its functions.

Several recent studies have attempted to explore the m^6^Am locations and functions, though some of the findings arising from these studies point to opposing conclusions. One study has implicated m^6^Am in regulating mRNA stability^3^, whereas another study did not find such an effect^20^. The two studies also suggested m^6^Am may be associated with increased translation efficiency based on correlations with external ribosome profiling data sources. Recently, several independent groups have identified the methyltransferase which catalyzes m^6^Am installation—*Pcif1*, and knocked it out in order to explore the m^6^Am functions^21–25^. In doing so, Akichika et al. findings suggested that m^6^Am *promotes* translation^22^. However, Sendinc et al. findings suggest that m^6^Am shows rather *reduced* translation^23^; whilst Boulias et al., and Raman Pandey et al., suggest rather it has an effect on mRNA stability^24,25^, along with an identified reduced body-weight in KO mice^25^. A possible reason for the discrepancy between the observed m^6^Am functions in *Pcif1* KO studies is that these methods relay on identifying missing m^6^A peaks in KO samples as the criterion for m^6^Am identification. Yet, missing m^6^A peaks could essentially still include some original m^6^A sites that are absent from other reasons—such as indirect effects associated with the bulk knockout stress, or even general quality variability between samples. While this procedure does provide clues for potential m^6^Am candidate sites, here, we identify a completely unconfounded pool of m^6^Am sites based on sites recognized by m^6^A-seq in mouse embryonic stem cells (mESCs) in which *Mettl3*, the main m^6^A methyltransferase—is KO^8^. Since these cells are completely depleted from m^6^A in their mRNA^8^, we are able to deduce true m^6^Am peaks, and thereby uncover reliably its association with increased mRNA stability, translation efficiency, and protein expression. Furthermore, our examination of m^6^A and m^6^Am profiles in HFD and ob/ob mice, uncover the dynamic role of the m^6^Am modification in regulating obesity, showing clear metabolic involvement of altered methylated genes, including two fatty acid-binding proteins *Fabp2*, *Fabp5*. In addition, we identify that FTO is overexpressed in fat mice, and importantly downregulates its m^6^Am gene targets. These results strongly implicate a significant role for m^6^Am in dynamic translation regulation of obesity.

## Results

### Identifying mRNA m^6^Am in m^6^A-depleted mESCs strongly establishes an association of m^6^Am with higher protein levels, translation efficiency, and mRNA stability

We deduced the m^6^Am profile from m^6^A sites mapped in *Mettl3* KO mESCs devoid entirely of mRNA m^6^A^8^ and identified m^6^Am peaks within 1,848 genes, of which 77% were detected within a window of up to 50 nucleotides from a known annotated TSS (Fig. 1c and Supplementary Data 1). In these reliable m^6^Am peaks we identified a robust correlation between the peak score (see “Methods”) and the percentage of adenosines at the annotated TSS (Fig. 1d, Pearson *r* = 0.82). Yet, given the considerable variability of TSS locations^17–19^, it is likely that in this m^6^A depleted sample non-adenosine annotated TSS peaks represent m^6^Am signals derived from an alternative A—TSS in its vicinity. Similarly, the few remaining peaks that were located >50 nucleotides of a known TSS, could still represent in part unidentified tissue-specific alternative TSS locations (as well as some background noise). As expected, collapsing all the DNA sequences of the peaks preceding and following known TSSs have identified the canonical BCA genomic consensus in addition to a bias towards pyrimidines in position −1 and a preceding TATA box, which are two hallmarks of transcription initiation^17^, (Fig. 1e).

Importantly, the use of cells devoid of m^6^A in mRNA enables the unequivocal identification of m^6^Am peaks and thus to reliably explore their functions. Testing the mRNA levels of m^6^Am-decorated genes in *Mettl3* KO mESCs^8^ revealed that these tend to be substantially more expressed than non-m^6^Am genes (Fig. 1f), even after removing genes that were decorated with m^6^A outside the 5′ UTR segment in wild type (WT) mESCs, (Supplementary Fig. 1a). A similar pattern of expression of m^6^Am modified genes was evident in WT mESCs, contrasting the inverse U-shaped expression pattern of m^6^A modified genes (Supplementary Fig. 1b). Notably, analysis of our previously published ribosome profiling data of the very same m^6^A-depleted samples^8^ revealed that m^6^Am genes tend to have higher ribosome footprint coverage even after controlling for their corresponding mRNA expression levels (Fig. 1g, *p* = 2.02 × 10^−35^, ANCOVA), as well as after excluding genes decorated with m^6^A in WT mESCs, (*p* = 0.046, ANCOVA), indicating higher translation efficiency. In addition, m^6^Am genes had higher ribosome release scores (RRS) that are associated with higher translation rates^26^ (Fig. 1h, *p* = 1.47 × 10^−7^, Mann Whitney), even after excluding m^6^A genes identified in WT mESCs (*p* = 0.012, Mann Whitney). Most importantly, high throughput proteomic profiling analyses showed that these effects were translated to significantly higher protein levels of m^6^Am- compared to non-m^6^Am-modified genes (Fig. 1i, *p* = 4.32 × 10^−33^, Mann Whitney) even after adjustment for their respective mRNA expression levels (Supplementary Fig. 1c, *p* = 6.19 × 10^−11^, ANCOVA); or upon excluding m^6^A-modified genes identified in WT mESCs (*p* = 7.4 × 10^−4^, ANCOVA). An identical pattern of higher protein levels of m^6^Am-modified genes was observed in WT mESCs controls (Supplementary Fig. 1d, *p* = 1.39 × 10^−6^, ANCOVA), contrasting the opposite pattern of reduced protein levels of m^6^A-modified genes (Supplementary Fig. 1e, *p* = 6.17 × 10^−8^, ANCOVA). Finally, analysis of the mRNA half-life rates of these m^6^Am-modified transcripts established that they exhibit higher mRNA stability than non-m^6^Am-decorated genes (Fig. 1j, *p* = *5.77* × 10^−36^, Mann Whitney); even after excluding m^6^A-methylated genes that were identified in WT mESCs (*p* = 6.9 × 10^−11^, Mann Whitney).

Our approach examines well-annotated mouse m^6^Am sites, free from m^6^A background, and in doing so enables the clear correlation of m^6^Am with higher protein levels, translation efficiency, and mRNA stability. Yet, since the comparisons are made between m^6^Am-marked genes versus *different* genes that are not methylated, the findings described here, and in previous studies, are still correlative. In order to examine if m^6^Am has a role in promoting these observed effects, it is necessary to capture the effects triggered by dynamic changes in m^6^Am deposition within the same genes, which lose or gain m^6^Am methylation through experimental perturbations. For this purpose and in order to explore the possible metabolic effects and dynamics of m^6^Am in-vivo, we explored its profile and functions following high fat diet in a mouse model. However, given the fact that *Mettl3* KO is embryonic lethal^8^ and normal mice mRNAs are also m^6^A methylated, we devised a more stringent approach for m^6^Am peak definition. Based on analysis of m^6^Am peak distribution in *Mettl3* KO cells, we considered only read ends falling within 25 nucleotides of a reported TSS site, with over 5 reads in IP, over two-fold change (IP/Input) and an FDR ≤5%. Applying these more stringent criteria to the same *Mettl3* KO mESCs mapping data^8^ resulted in the identification of robust peaks in 849 genes. The analysis of this highly stringent peak dataset recapitulated all the aforementioned results regarding association with mRNA stability, translation efficiency and mRNA and protein levels. Finally, to assess the false positive rate of our approach we reversed the IP with the Input samples. This control analysis resulted in only 3.5% (30) peaks, which indicates a very low level of false positive peaks.

### m^6^Am- rather than m^6^A-decorated genes are enriched in metabolic processes annotation

**Fig. 2:**
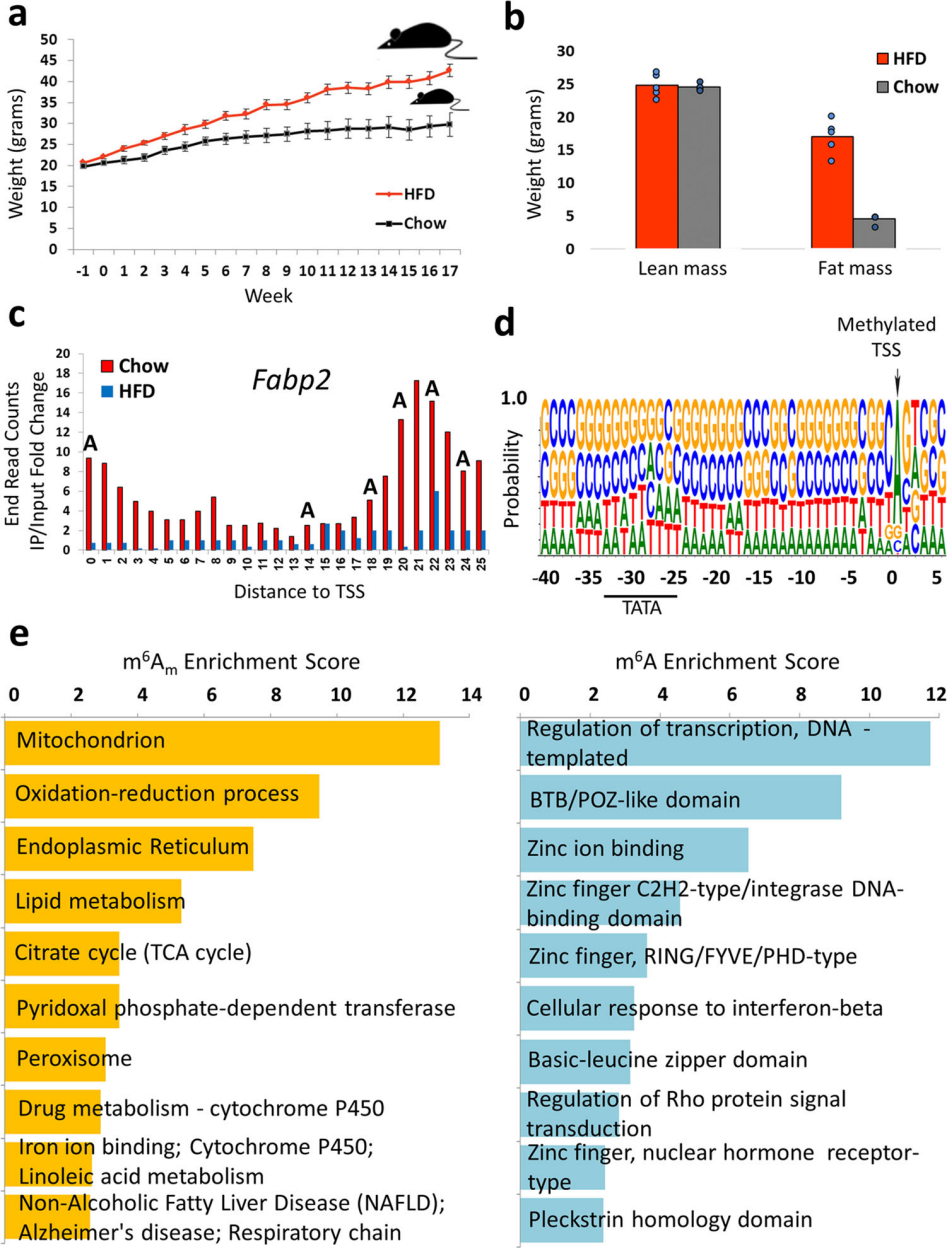
High fat diet mice physiologic parameters and enrichment of m^6^Am-modified genes in GO terms associated with metabolic processes. **a** Gain in weight of mice fed a high fat diet (HFD) or regular chow diet across weeks. *N* = 10 (5 HDF mice, and 5 Chow lean control mice), error bars denote SEM. **b** NMR measurement of lean and fat mass body composition, indicating that the gain in weight was mainly in fat mass. *N* = 10 (5 HDF mice, and 5 Chow lean control mice). **c** Fold change of read end counts (IP/Input) within a sliding window of 5 base-pairs around a reported adenosine TSS in the *Fabp2* gene in lean (chow) and HFD mice. All adenosines in the sequence are indicated. **d** Sequence logo of the m^6^Am peaks around and upstream the annotated TSS within the DNA, portraying the canonical m^6^Am genomic consensus. **e**. Gene ontology analysis of m^6^Am and CDS m^6^A (>50 nt of TSS) showing a clear enrichment of m^6^Am- but not in m^6^A-modified genes, in GO terms associated with metabolic processes. Source data are provided as a Source Data file.

In order to explore the m^6^A and m^6^Am deposition dynamics and functions in an obesity-related perturbation, we investigated the liver m^6^Am and m^6^A methylomes in response to high fat Western-diet (HFD) stress perturbation. Four weeks old mice were divided into two groups of 5 mice each, feeding on either standard chow diet or HFD for 17 weeks *ad libitum*. Mice fed on HFD gained significantly more weight (weighing a mean of 43 grams at the end of the experiment) than mice that were fed regular chow diet (mean of 30 grams at the end of the experiment, *p* = 0.008 (Fig. 2a). Consequently, the liver of HFD mice weighed more (mean of 2.19 grams) than that of standard chow diet mice (mean of 1.76 grams, *p* = 0.05, Supplementary Fig. 2a). In addition, an NMR analysis of the weight distribution indicated that the difference in weight was mainly in fat mass rather than in muscle mass (Fig. 2b). As expected, blood glucose levels of HFD mice after 6 h of fasting were higher (135 µg/dL) than control mice (109 µg/dL, *p* = 0.01, Supplementary Fig. 2b).

A high throughput analysis of m^6^Am and m^6^A peaks in lean vs. fat mice liver, detected m^6^Am peaks (identified in at least 2 of 3 mice in each group) in 1,176 genes in lean mice (calculated based on read ends falling within the first 25nt of reported TSS as described above) and m^6^Am peaks in 607 genes in fat mice (Supplementary Data 2). The majority of peaks (70%) were either lean or fat specific, while only 30% were common to both groups. An indicative example of an exclusively lean-specific m^6^Am peak within a reported alternative A-TSS of the *Fabp2* gene is provided in Fig. 2c. We also detected m^6^A peaks in 2,727 genes in lean mice (starting > 50 nt of reported TSS, beyond 5’ UTR regions) and in 2,233 genes in fat mice (Supplementary Data 3). Notably, a significant percentage of m^6^A peaks (52%) were common to both lean and fat mice with the remaining 48% specific to either lean or fat mice. Importantly, collapsing the identified m^6^Am peaks preceding and following the annotated TSS within the DNA, identified the canonical m^6^Am BCA genomic consensus (Fig. 2d). In contrast, analyzing the mRNA sequences preceding and following non-5′UTR m^6^A peaks identified the canonical (RRACH) m^6^A consensus (Supplementary Fig. 3a). Analyzing the mRNA expression profiles demonstrated higher expression levels of m^6^Am modified genes, and revealed an inverse U-shaped expression profile of m^6^A modified genes (Supplementary Fig. 3b). In order to assess the dominant processes associated with m^6^Am or m^6^A methylated genes, we performed a gene ontology analysis. Although this analysis is very broad, we remarkably discovered that generally m^6^Am-decorated genes across lean or obese mice are predominantly enriched in multiple obesity and metabolic-related processes, while m^6^A genes did not seem to clearly display significant enrichment for metabolic processes (Fig. 2e; Supplementary Fig. 3c). Similar metabolic pathways were identified when we analyzed m^6^Am-modifed genes discovered in published *Pcif1* KO studies (Supplementary Table 1)^22–25^. Similarly, the lists of genes with differentially methylated peaks in lean vs. fat mice showed mainly m^6^Am but not m^6^A enrichment in metabolic and obesity-related processes (see Supplementary Data 2, 3). Hence, in subsequent analyses, we focused primarily on the effects observed in differentially m^6^Am-modified gene transcripts.

### m^6^Am regulates mRNA and protein levels in response to HFD

**Fig. 3:**
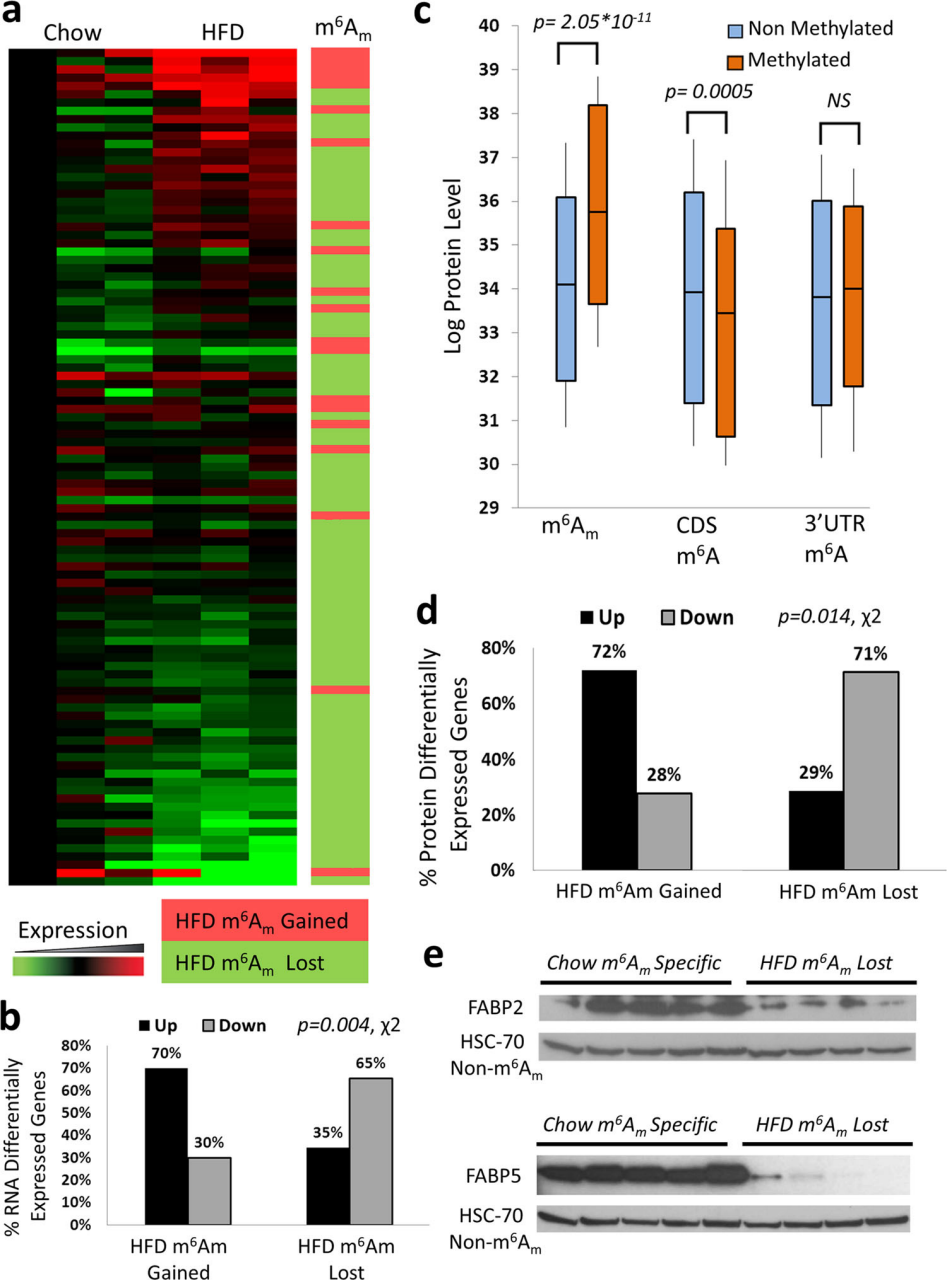
m^6^Am significantly regulates mRNA and protein levels upon high fat diet. **a** mRNA expression heatmap of >1.5 fold differentially expressed genes with differential m^6^Am methylation in HFD. Heatmap colors represent differential mRNA expression fold-change levels in the respective gene between mice samples (green - lower expression; red - higher expression). Right panel indicates if m^6^Am was gained (light red - HFD specific) or lost (light green - chow specific) in HFD. **b** Heatmap quantification of the mRNA differentially expressed genes that gained or lost m^6^Am upon HFD. Two-tailed *p*-value of the Chi-square test is reported. **c** Log protein expression levels of m^6^Am or m^6^A methylated genes versus non-methylated genes in lean control (chow) mice. Two-tailed *p*-values of the Mann Whitney U test are reported, covering 2246, 1998, and 5737 genes with the respective locations. Box plots surround the 1–3 quartiles, whiskers denote 1.5 interquartile range. **d** Full proteomic profiling proportions of genes with protein differential expression >1.5 fold, which gained or lost m^6^Am upon HFD. Two-tailed *p*-value of the Chi-square test is reported. Full proteomic abundance profiling was conducted on two HFD mice samples and two lean chow control mice samples covering a total of 5914 proteins detected. **e** Western blot of Fabp2 and Fabp5 genes, which were found decorated with m^6^Am only in lean mice. The results show a clear repeating pattern of overexpression across all lean biological replicates (*N* = 5) versus all fat biological replicates (*N* = 4). See Supplementary Fig. 4c for the quantification of these signals. The available molecular weight markers can be seen within the full scan blots which are available as Source data accompanying this figure, provided as a Source Data file.

In order to test the effects and functions of diet-related m^6^Am dynamics, we looked at genes with differential methylation which have gained or lost m^6^Am in HFD fed mice. We first looked at mRNA expression levels of differentially expressed genes in lean and fat mice by RNA-seq. This differentially expressed list of genes included only genes that either gained or lost m^6^Am, and that were also >1.5-fold differentially expressed, thereby focused on a total of 101 genes. We found that among genes that gained m^6^Am there were a significantly higher fraction of upregulated transcripts than expected, while genes that lost m^6^Am in fat mice had a significantly higher fraction of downregulated genes than expected (Fig. 3a,b, *p* = 0.004, χ2). In order to test if this effect is observed also at the protein level, we performed a high throughput proteomic profiling. Again, we found that m^6^Am-decorated genes are associated with higher protein levels than non-m^6^Am-modified genes (Fig. 3c). This association was in accordance with diet-related differential m^6^Am methylation patterns as well. Genes that gained m^6^Am in HFD fed mice displayed higher fraction of upregulated genes than expected, while genes that lost m^6^Am displayed a higher fraction of downregulated genes than expected (Fig. 3d, *p* = 0.014, χ2), suggesting a role for m^6^Am decoration in the regulation of gene expression. While the effect on the protein level presented here is likely augmented by the effect observed at the RNA level, removing all the above >1.5-fold differentially RNA expressed genes, preserves this pattern of results (Supplementary Fig. 4a). Similarly, we observed that m^6^Am-modified genes consistently display higher protein levels than non-m^6^Am-modified genes, even after controlling for their RNA expression level (Supplementary Fig. 4b). For example, two obesity-related fatty acid-binding proteins, FABP2 and FABP5, which lost their m^6^Am under HFD were both significantly downregulated at the protein levels (Fig. 3e and Supplementary Fig. 4c), while *Fabp5* also displayed >10-fold downregulation at the mRNA level as well.

### FTO is upregulated in HFD and its cellular perturbation affects protein levels of m^6^Am targets

**Fig. 4:**
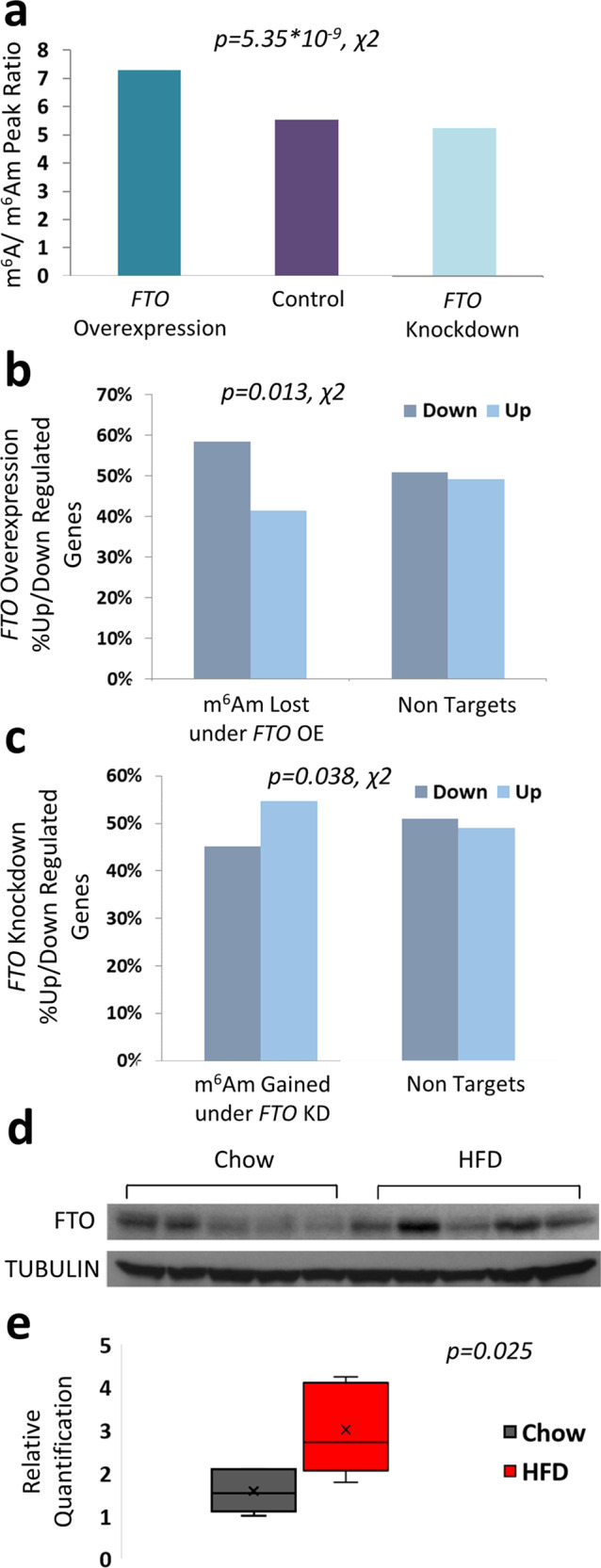
FTO is highly expressed in HFD mice and its cellular perturbation affects protein levels of its m^6^Am-modified targets. **a** m^6^A/m^6^Am peak ratio in *FTO* overexpression, *FTO* knockdown, and control HepG2 cells. Two-tailed *p*-value of the Chi-square test is reported. **b** Full proteomic abundance profiling of HepG2 cells overexpressing *FTO*. Results display a higher fraction of downregulated genes that lost m^6^Am in comparison to non-targets. Two-tailed *p*-value of the Chi-square test is reported. **c** High-throughput proteomic profiling of *FTO* knockdown in HepG2 cells. Results display a higher fraction of upregulated genes that gained m^6^Am in comparison to non-targets. Two-tailed *p*-value of the Chi-square test is reported. Full proteomic abundance profiling was conducted on an HepG2 *FTO* overexpression sample, an HepG2 *FTO* Knockdown sample, and an HepG2 control sample, and covered a total of 5487 proteins detected. **d** Western blot of liver FTO in HFD and regular chow diet control mice showing higher expression levels of FTO in HFD mice relative to the normalizing TUBULIN. The results show a clear repeating pattern of overexpression through most HFD biological replicates versus most lean control biological replicates. The available molecular weight markers can be seen within the full scan blots, which are available as source data. **e** Quantification of the FTO western blot signal relative to normalizing TUBULIN. Box plot surrounds the 1–3 quartiles, whiskers denote 1.5 interquartile range. *p*-values are indicated (two-tailed student t-test), *N* = 10 (five HFD and five Chow lean controls). Source data are provided as a Source Data file.

Next, we wished to test whether *FTO* has a preference towards demethylating m^6^Am rather than m^6^A as was recently reported^3^. We, therefore, overexpressed, as well as knocked-down *FTO* in human hepatocarcinoma cell line, HepG2 cells, and compared the number of m^6^A and m^6^Am peaks relative to control cells. Overall, we detected considerably fewer peaks in the overexpressing cells (522 m^6^Am and 3,803 m^6^A out of 4,325 peaks) than in the control cells (1,142 m^6^Am, and 6,316 m^6^A out of 7,458 peaks) and in the knockdown cells, respectively (1,672 m^6^Am, and 8,998 m^6^A out of 10,670 peaks, see Supplementary Data 4). The ratio of m^6^A/m^6^Am in control cells and knockdown cells was lower (5.5 fold, and 5.3 fold respectively), while in *FTO* overexpressing cells this ratio was significantly higher (7.3 fold, *p* = 5.35 × 10^−9^, χ2) (Fig. 4a), indicating that *FTO* affected both modifications with a slight preference towards demethylating m^6^Am. To further explore if differential m^6^Am methylation under *FTO* perturbation has a role in regulating protein levels, we performed high-throughput proteomic profiling. We observed that *FTO* targets which lost their m^6^Am peak under *FTO* overexpression were overly downregulated (Fig. 4b). Conversely, newly identified m^6^Am peaks under *FTO* knockdown showed rather increased prevalence of genes with protein levels higher than expected (Fig. 4c). In order to further assess if *Fto* has a specific role in HFD, we analyzed its expression in HFD fed mice. Western blot analysis of FTO in the liver of HFD fed mice showed that it was significantly overexpressed compared to the lean chow diet control mice (Fig. 4d, e), suggesting that the enzyme may have a dynamic regulatory role in demethylating m^6^Am (and m^6^A) under high fat diet.

### Analysis of ob/ob mice suggests a similar regulation pattern of m^6^Am and demonstrates higher expression levels of FTO

**Fig. 5:**
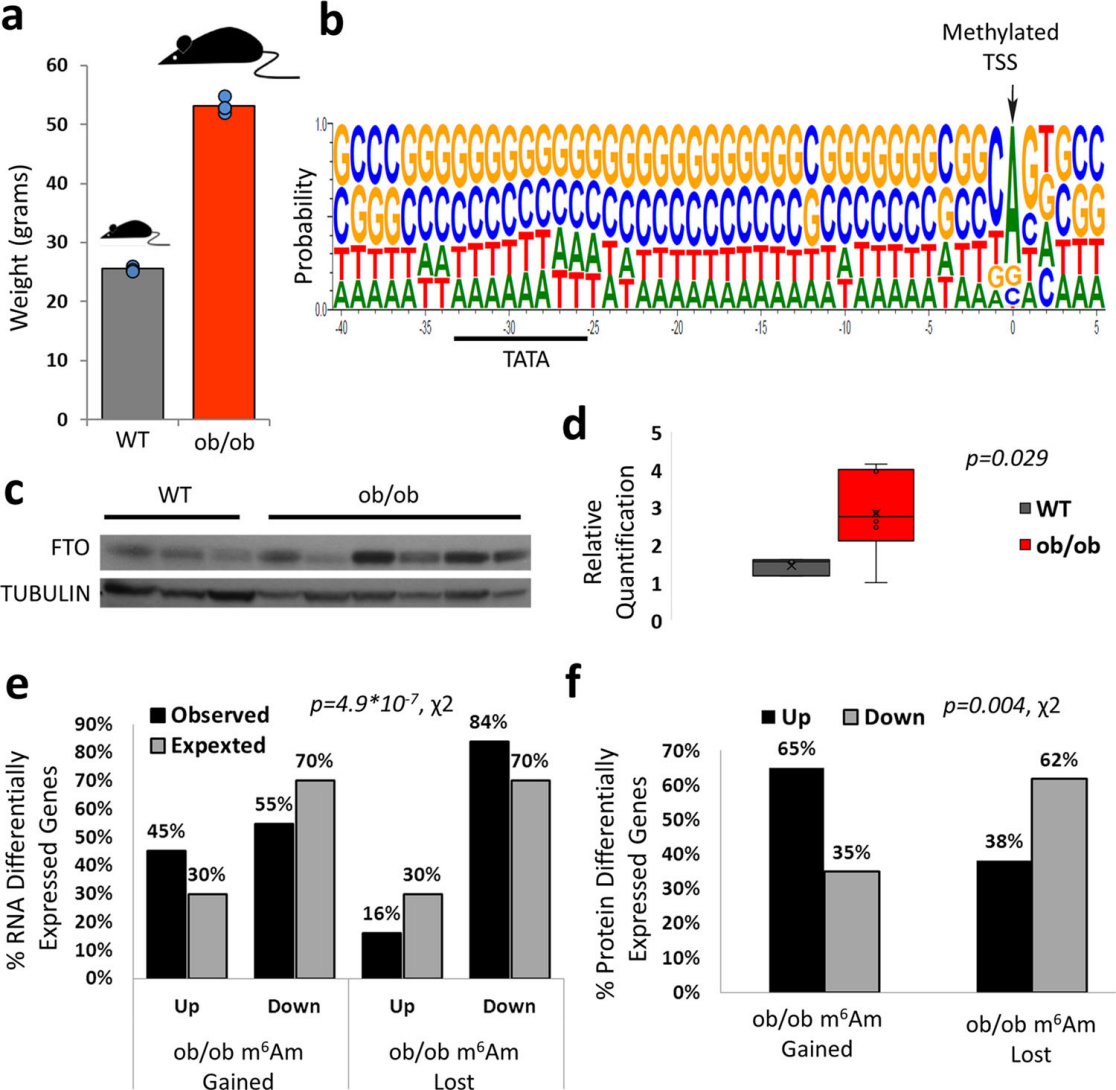
m^6^Am regulates mRNA and protein expression in ob/ob mice. **a** ob/ob mice and their WT littermates’ weight on the day of sacrifice. *N* = 9 (4 ob/ob mice and 3 WT mice). **b** Sequence collapse of the m^6^Am peaks around and upstream the annotated TSS within the DNA portraying the canonical m^6^Am genomic consensus. **c** Western blot of FTO in ob/ob and WT mouse livers relative to the normalizing TUBULIN. The results show a clear repeating pattern of overexpression across five of six ob/ob biological replicates versus all three lean control biological replicates. The available molecular weight markers can be seen within the full scan blots, which are available as source data. **d** Quantification of the FTO western blot signal relative to the normalizing TUBULIN. Box plot surrounds the 1–3 quartiles, whiskers denote 1.5 interquartile range, *p*-values are indicated (two-tailed student t-test with unequal variance), *N* = 9 (six ob/ob and three WT lean controls). **e** Proportions of genes which were upregulated or downregulated (>1.5 fold) upon gain or loss of m^6^Am in ob/ob mice, relative to their overall expected ratios in the sample without m^6^Am peak categorization. Two-tailed *p*-value of the Chi-square test is reported. **f** Full proteomic abundance profiling proportions of genes with protein differential expression >1.5 fold which gained or lost m^6^Am in ob/ob mice. Two-tailed *p*-value of the Chi-square test is reported. Full proteomic abundance profiling was conducted on two ob/ob mice samples and two lean wt samples covering a total of 5568 proteins detected. Source data are provided as a Source Data file.

In order to substantiate the regulatory effects of m^6^Am in yet another in vivo perturbation of obesity, we performed a corresponding high throughput methylation analysis in ob/ob mice. ob/ob mice genetically lack leptin receptor, and as a result, eat excessively without reaching satiety. Both ob/ob mice and control WT littermates were feeding on the same diet but the ob/ob weighed almost double than the controls (mean of 53 vs. 26 grams, Fig. 5a). In high throughput m^6^Am methylation profiling, we similarly detected the typical BCA genomic consensus and an upstream TATA box (Fig. 5b), (for the non-5′ UTR m^6^A peaks RNA consensus see Supplementary Fig. 5a). Most notably, both *Fabp2* and *Fabp5* were uniquely m^6^Am methylated in WT lean mice, but not in fat ob/ob mice, a pattern that was also detected in the HFD fed mice. Again, we observed that the majority of m^6^Am peaks (63%) were either lean or fat specific, whereas most m^6^A peaks (62%) were peaks common to both lean and fat mice (for the full m^6^Am and m^6^A methylation maps see Supplementary Data 5, 6). Similarly, we identified higher expression levels of FTO in ob/ob mice than in WT controls (Fig. 5c, d). In order to examine if m^6^Am has comparable regulation properties in ob/ob mice, we examined the differential contribution of m^6^Am to mRNA levels by RNA-seq. Again, we found that genes which lost m^6^Am methylation in ob/ob mice, had a higher fraction of downregulated genes than expected, while among genes that gained m^6^Am methylation, a higher-than-expected fraction was upregulated (Fig. 5e). To complement this analysis, we performed high throughput proteomic profiling and observed a similar regulation pattern at the protein level (Fig. 5f) further supporting the regulatory contribution of m^6^Am to translation. In accordance, overall m^6^Am modified genes displayed consistently higher protein levels than non-m^6^Am modified genes, even after adjusting for their RNA expression level (Supplementary Fig. 5b).

## Discussion

In this study, we explored both the m^6^Am and m^6^A liver methylation profile dynamics and functions in mice under HFD and in ob/ob mice. We present data linking the m^6^Am modification with dynamic functional regulation of obesity. Gene ontology (GO) analysis revealed that m^6^Am- but not m^6^A-methylated transcripts were highly enriched in GO terms of metabolic processes. Additionally, differential removal of the m^6^Am modification in fat mice was associated with downregulation of protein and mRNA transcript levels, and correspondingly, upregulation of protein and mRNA expression upon m^6^Am addition. We identified differentially m^6^Am methylated key metabolic genes in lean and obese mice including the two m^6^Am lean specific methylated fatty acid-binding proteins *Fabp2* and *Fabp5*. Most notably, the silencing of *Fabp2* is known for its contribution to weight gain in male mice^27^. Similarly, *Fabp5* is a pivotal metabolic gene whose silencing is implicated in preserving healthier metabolic parameters including insulin sensitivity^28^. *Fabp5* was similarly identified as an m^6^Am-methylated gene transcript^24^. We further observed that FTO is noticeably upregulated in fat mice and its cellular overexpression and knockdown correspondingly affect the protein levels of its m^6^Am-methylated targets. Most notably, the same pattern of results was recapitulated in two complementing in-vivo perturbations of obesity—HFD and in ob/ob overfeeding mice. Lastly, by analyzing the methylation profile of mESCs depleted entirely of m^6^A mRNA background via *Mettl3* KO, we deduced m^6^Am sites in mRNA and their role in translation regulation in a controlled un-confounded way, and generated a list of confirmed mammalian m^6^Am-methylated genes. This analysis of unequivocal m^6^Am sites identified that capped m^6^Am genes have higher protein and mRNA levels, increased mRNA stability, ribosome footprints, and ribosome release scores (RRS). These results strongly establish the role of m^6^Am in translation regulation, and inform the unresolved debate on its functions^21–25^.

Importantly, the m^6^A/m eraser, *FTO*, is considered one of the genes that are most robustly associated with human obesity based on single nucleotide polymorphism (SNP) in one of its introns^1^. While *Fto* overexpression in mice was shown to actively promote obesity^29^, affect hepatic leptin-stat3-glucose signaling^30^, and conversely protect from obesity in an *Fto* KO mouse model^31^, the nature of this association is still not clearly defined. It is also noteworthy that the expression of *Fto* was shown to be dynamically regulated in various obesity models and tissues. For example, it was observed to be downregulated in adipose tissue in HFD mice^32^ and in mouse liver following insulin treatment^33^; or upregulated in the liver of rats with Nonalcoholic Fatty Liver Disease^34^ and in mouse liver of fasting mice^35^; but notably, observed in this study as well to be significantly upregulated in the liver of both HFD and ob/ob overfeeding mice. These findings highlight the significance of a dynamic regulation of *Fto* expression in metabolic tissues and specifically in the liver. While *Fto* expression is one important layer of regulation for m^6^Am removal, it is possible that additional, yet unknown, erasers and writers are at play and further modulate m^6^Am deposition. These players may also participate in the cases where m^6^Am was detected only in fat mice. Note that in our data, we did not observe meaningful differences in the expression of the recently identified m^6^Am writer *Pcif1*, yet a small 1.39-fold increase in its mRNA expression was observed in HFD.

Despite the clear associations and expression dynamics of *FTO* in obesity, to date, little is known about the contributions and interplay between *FTO*’s two target modifications—m^6^A, and m^6^Am in affecting metabolism. The current work significantly promotes our understanding of the mRNA modification metabolic effects by analyzing both of the mRNA modification targets of *FTO* (m^6^A and m^6^Am) in two different in vivo obesity mouse models. Our findings support a more dominant metabolic contribution of m^6^Am rather than m^6^A in mediating metabolic effects under HFD and in ob/ob overfeeding mice. In addition, the identification of lean-specific m^6^Am-methylated key metabolic genes (including *Fabp2*, and *Fabp5*) further support m^6^Am function in the dynamic regulation of obesity. Importantly, the higher-than-expected fraction of downregulated genes that lost m^6^Am, suggests a significant involvement of m^6^Am in the dynamic regulation of protein and mRNA expression in obesity. In addition, the direct analysis of unequivocal m^6^Am targets in cells deprived of m^6^A background implies that the putative mechanisms of action of m^6^Am involve increased mRNA half-life, but also increased translation efficiency.

In this study, we found that m^6^Am-modified transcripts are enriched in GO terms of metabolic processes while m^6^A-modified transcripts do not exhibit a clear enrichment. This suggests that the m^6^Am modification may have a dominant role in obesity regulation. Yet, our findings do not rule out the possibility of additional roles of *FTO* that can also be mediated by m^6^A (e.g.,^4^^,^^5^), since both modifications were reported to be valid substrates of *FTO*^2^^,^^3^. While we found only a small subset of obesity metabolic genes that were differentially m^6^A methylated, it is possible that some of these genes have a yet unknown metabolic function.

RNA epitranscriptomics is a rapidly growing field that unveiled dramatic revelations of various mRNA modifications in affecting gene regulation and multiple cellular functions including alternative splicing (e.g.,^12^^,^^8^), stability^36^, and translation initiation^9^. The current work highlights the dynamic involvement of the m^6^Am modification in a disease state of obesity and in metabolic regulation. In particular, we also strictly support these observed functions of m^6^Am by validating them in pure experimental conditions devoid of the confounding effects of m^6^A background, a critical issue that is likely a significant source for the continuous disagreement on its postulated functions^21–25^. Most notably, our findings demonstrate that m^6^Am is dynamically regulated in-vivo in response to high fat Western diet, and in response to excessive eating in ob/ob mice, thus enabling significant flexibility that is vital to changing metabolic environments^37^. Taking together, our findings strongly support a significant new layer of dynamic functional regulation in obesity by the m^6^Am mRNA modification.

## Methods

### Cell lines, cultures, and treatments

Human hepatocellular carcinoma cell line, HepG2 (ATTC HB-8065), was maintained in DMEM (Gibco, Invitrogen) containing 4.5 g/l glucose and L-glutamine supplemented with 10% FBS and penicillin/streptomycin. Cells were routinely checked for mycoplasma contamination and tested negative. Where indicated, HepG2 cells were transfected with a human *FTO* gene plasmid that was received from^2^ and is based on the mammalian vector pcDNA3 (Invitrogen) with N-terminal FLAG-tag. The *FTO* knockdown siRNA sequence was [AAAUAGCCGCUGCUUGUGAGA] (Sigma). Transient transfections were performed using the Lipofectamine reagent (Lipofectamine 2000 and Lipofectamine RNAimax, Invitrogen, USA) according to the manufacturer’s instruction. *Mettl3* knockout mESCs and WT control mESCs were obtained from^8^.

### Animal backgrounds and treatments

Animal rooms were maintained at 21–24 °C and 35–75% relative humidity, with 12/12 h (7 a.m. to 7 p.m.) dark–light cycle. Cages were routinely replaced every 10–14 days. We have complied with all relevant ethical regulations for animal testing and research. All experiments received ethical approval and were conducted in accordance with the Institutional Animal Care and Use Committee at Bar Ilan University.

### High fat diet fed mice

Four weeks old male C57BL/6 mice were kept under specific pathogen-free and 12 h day/night conditions and fed a standard chow diet (24 kcal% Protein, 65 kcal% Carbohydrate,11 kcal% Fat, Altromin 1324 tpf) for the first 2 weeks, until 6 weeks of age. The mice were then randomly divided into two diet groups of 5 mice each. Mice were fed with a standard chow diet for an additional 17 weeks, and in the HFD group were fed high fat diet (17 kcal% Protein, 23 kcal% Carbohydrate,60 kcal% Fat, Altromin C-1090-60). All groups were fed *ad libitum*. Following this period, the mice were sacrificed and tissues harvested, snap-frozen in liquid nitrogen, and stored in −80 °C until further use. 3 different HFD biological replicate mice liver tissues and 3 controls were used for m^6^A/m^6^Am-seq analysis.

### Ob/ob mice

Ob/ob and WT homozygotes/heterozygotes littermates mice were of C57BL/6 background. Liver tissues from 3 different ob/ob biological replicate mice (13 weeks-old; 2 males, 1 female) and 3 WT (13 weeks-old; 2 males, 1 female) mice controls were used for m^6^A/m^6^Am-seq analysis. Additional 3 ob/ob mice (1 male, 13 weeks-old; and 2 females 36 weeks-old) were used for the extended western blot.

### Blood parameters

Blood glucose was measured from samples obtained by tail bleeding after 6 h fasting. Glucose levels were tested using Ascensia Elite glucose meter (Bayer, Leverkusen, Germany).

### NMR analysis

Mice were scanned using the Minispec Analyzer (Bruker Minispec LF50), which measures lean and fat composition.

### RNA purification

Total RNA from cells in culture and mouse tissues was purified using PerfectPure RNA Cultured Cell Kit (5′ Prime) and PerfectPure RNA Tissue Kit (5′ Prime), respectively, and DNase-treated. RNA integrity was evaluated on Bioanalyzer (Agilent 2100 Bioanalyzer), requiring a minimal RNA integrity number (RIN) of 8.5. Experiments were conducted in biological replicates.

### m^6^A/m^6^Am-seq

Total RNA (>300 µg) of samples were chemically fragmented into ~100-nucleotide-long fragments by 5 min incubation at 94 °C in fragmentation buffer (10 mM ZnCl2, 10 mM Tris-HCl, pH 7). The fragmentation reaction was stopped with 0.05 M EDTA, followed by standard ethanol precipitation. Samples were resuspended in H_2_O at ~1 mg ml/1 concentration and subjected to m^6^A-seq. Fragmented RNA was incubated for 2 h at 4 °C with 5 mg of affinity-purified anti-m^6^A polyclonal antibody (Synaptic Systems cat. no. 202 003) in IPP buffer (150 mM NaCl, 0.1% NP-40, 10 mM Tris-HCl, pH 7.4). The mixture was then immunoprecipitated with protein-A beads (Repligen) at 4 °C for an additional 2 h. After extensive washing, bound RNA was eluted from the beads with 0.5 mg ml/1 N^6^-methyladenosine (Sigma-Aldrich) in IPP buffer, and ethanol precipitated. RNA was resuspended in H_2_O and used for library generation with the NEBNext Ultra RNA Library Prep Kit for Illumina (New England Biolabs). Sequencing was carried out on Illumina HiSeq2500 according to the manufacturer’s instructions, using 10 pM template per sample for cluster generation, and sequencing kit V2 (Illumina). These raw data are available at NCBI SRA series PRJNA701370 (public release date set to 2021-08-01).

### Peak calling

Adaptors and low-quality bases were trimmed from raw sequencing reads using cutadapt^38^. Reads were aligned to the relevant genome (human-hg19, mouse-mm10, using Tophat2 (version 2.0.12)^39^. Non-unique reads mapping to more than five locations were discarded from downstream analysis. For m^6^A peaks, enrichment of immunoprecipitation over input experiments were identified using MACS2 (version 2.1.0.20140616)^40^. MACS2-identified peaks were intersected with a database of exons of the relevant genome (Ensemble annotation). Only peaks identified (FC ≥ 2, FDR ≤ 0.05) were considered. Negative peaks as a control were identified by switching the immunoprecipitation and input samples.

### Identifying m^6^Am sites

Since m^6^Am occurs only in transcription start sites (TSSs), we downloaded the genomic position of TSSs from Refseq (PMID: 24259432), UCSC known genes (PMID: 16500937), GENECODE Version M14 (Ensembl 89; PMID: 16925838), and EPD (PMID: 25378343). In each of the aligned samples, we counted the number of 5′ read ends that resides within 25 bp intervals downstream of the TSS. In order to avoid PCR duplicates we allowed a maximum of 5 duplicate reads ending in a single bp position. For each TSS we calculated the fold change as follows:

$$\frac{{{{{{{{\rm{Number}}}}}}\,{{{{{\rm{Of}}}}}}\,5^{\prime} \,{{{{{\rm{reads}}}}}}\,{{{{{\rm{in}}}}}}\,{{{{{\rm{TSS}}}}}}\,{{{{{\rm{(IP)}}}}}}}}}{{{{{{\rm{Number}}}}}}\,{{{{{\rm{Of}}}}}}\,5^{\prime} \,{{{{{\rm{reads}}}}}\,{{{{{\rm{in}}}}}}\,{{{{{\rm{TSS}}}}}}\,{{{{{\rm{(input)}}}}}}}}\,*\, \frac{{{{{{\rm{Library}}}}}}\,{{{{{\rm{size}}}}}}\,{{{{{\rm{(input)}}}}}}}{{{{{{\rm{Library}}}}}}\,{{{{{\rm{size}}}}}}\,{{{{{\rm{(IP)}}}}}}}$$

Cases with zero input coverage within 100 bp downstream of the TSS (to account for the average length of RNA fragments) were filtered out. For each TSS *p-*value was calculated using fisher’s exact test followed by FDR correction. Only TSSs with over 5 reads in IP, including a fold change greater than 2 and FDR ≤ 0.05 (using Benjamini–Hochberg multiple testing adjustment), and that did not have an identified m^1^A peak in that location^9^ were kept for further analyses. In genes with more than one TSS we included the most significant TSS. For negative peak calculation as a control we performed the same approach but with using the IP as input and vice versa. Only peaks occurring in at least 2 of 3 mice were considered as m^6^Am methylated in the lean or fat mice groups.

### m^6^Am genomic consensus motif

For each TSS suspected to be m^6^Am methylated we retrieved the nucleotide genomic sequence 40 bp upstream and 5 bp downstream. The nucleotide probability logo was created using webLogo3 (PMID: 15173120) on all genes with a significant TSS peak.

### m^6^A MEME RNA consensus motif search

MACS-identified peaks with FDR#5% were sorted according to their fold change. The top 500 peaks falling within known genes were placed in de novo motif analysis. 101-nucleotide-long sequences derived from the sense strand and centered around the peak summit were used as input for MEME^41^.

### RNA expression levels

Fragments per kilobase of transcript per million mapped reads (FPKM) values were calculated by the CUFFLINKS tool (version 2.2.1)^42^. And differential expression analysis was done using the DESeq Bioconductor package as part of the Cufflinks package^43^. Features were considered as differentially expressed only when fold change >1.5.

### Gene ontology (GO) enrichment

m^6^Am or CDS (>50 bp) m^6^A Methylated genes in ≥2 mice were uploaded to DAVID Bioinformatics Resources (http://david.abcc.ncifcrf.gov) and functional annotation clustering enrichment analysis was performed using the default parameters with all adequately expressed genes as background. The resulting top 10 most enriched clusters were reported using the most occurring relevant terms.

### Proteomics

#### Sample preparation

Experiments were conducted in biological replicates. Cell pellets were lysed with 5% SDS in 50 mM Tris-HCl. Lysates were incubated at 96 °C for 5 min, followed by six cycles of 30 s of sonication (Bioruptor Pico, Diagenode, USA). Protein concentration was measured using the BCA assay (Thermo Scientific, USA) and a total of 100 μg protein was reduced with 5 mM dithiothreitol and alkylated with 10 mM iodoacetamide in the dark. Each sample was loaded onto S-Trap mini-columns (Protifi, USA) as described^44^. In brief, after loading, samples were washed with 90:10% methanol/50 mM ammonium bicarbonate. Samples were then digested with trypsin (1:50 trypsin/protein) for 1.5 h at 47 °C. The digested peptides were eluted using 50 mM ammonium bicarbonate; trypsin was added to this fraction and incubated overnight at 37 °C. Two more elutions were made using 0.2% formic acid and 0.2% formic acid in 50% acetonitrile. The three elutions were pooled together and vacuum-centrifuged to dry. Samples were kept at −80 °C until analysis.

#### Liquid chromatography

ULC/MS grade solvents were used for all chromatographic steps. Each sample was fractionated using high pH reversed-phase followed by low pH reversed-phase separation. 200 µ digested protein was loaded using high Performance Liquid Chromatography (Acquity H Class Bio, Waters, Milford MA, USA). Mobile phase was: (A) 20 mM ammonium formate pH 10.0, (B) acetonitrile. Peptides were separated on an XBridge C18 column (3×100mm, Waters) using the following gradient: 3% B for 2 min, linear gradient to 40% B in 50 min, 5 min to 95% B, maintained at 95% B for 5 min and then back to initial conditions. Peptides were fractionated into 15 fractions. The fractions were then pooled: 1 with 8, 2 with 9, 3 with 10, 4 with 11, 5 with 12, 6 with 13, and 7 with 14–15. Each fraction was vacuum dried, then reconstituted in 25 µL 97:3 acetonitrile: water + 0.1% formic acid. Each pooled fraction was loaded using split-less nano-Ultra Performance Liquid Chromatography (10 kpsi nanoAcquity; Waters, Milford, MA, USA). The mobile phase was: A) H_2_O + 0.1% formic acid and B) acetonitrile + 0.1% formic acid. Desalting of the samples was performed online using a reversed-phase Symmetry C18 trapping column (180 µm internal diameter, 20 mm length, 5 µm particle size; Waters). The peptides were then separated using a T3 HSS nano-column (75 µm internal diameter, 250 mm length, 1.8 µm particle size; Waters) at 0.35 µL/min. Peptides were eluted from the column into the mass spectrometer using the following gradient: 4 to 30%B in 150 min, 30 to 90%B in 5 min, maintained at 90% for 5 min, and then back to initial conditions.

#### Mass spectrometry

The nanoUPLC was coupled online through a nanoESI emitter (10 μm tip; New Objective; Woburn, MA, USA) to a quadrupole orbitrap mass spectrometer (Q Exactive HF, Thermo Scientific) using a FlexIon nanospray apparatus (Proxeon). Data were acquired in data-dependent acquisition (DDA) mode, using a Top20 method. MS1 resolution was set to 120,000 (at 200 m/z), mass range of 300–1650 m/z, AGC of 3e6, and maximum injection time was set to 20msec. MS2 resolution was set to 30,000, quadrupole isolation 1.7 m/z, AGC of 1e5, dynamic exclusion of 30 s, and maximum injection time of 60msec.

#### Data processing and analysis

Raw data were imported into the Expressionist^®^ software version 14 (Genedata, Switzerland) and processed as described in^45^. The software was used for retention time alignment and peak detection of precursor peptides. A master peak list was generated from all MS/MS events and sent for database searching using Mascot v2.5.1 (Matrix Sciences). Data were searched against the mouse or human sequences UniprotKB (http://www.uniprot.org/) appended with common laboratory contaminant proteins. The fixed modification was set to carbamidomethylation of cysteines and variable modifications were set to oxidation of methionines and deamidation of N or Q. Search results were then filtered using the PeptideProphet algorithm to achieve maximum false discovery rate of 1% at the protein level. Peptide identifications were imported back to Expressionist to annotate identified peaks. Quantification of proteins from the peptide data was performed using an in-house script. Data was normalized base on the total ion current. Protein abundance was obtained by summing the three most intense, unique peptides per protein. Protein abundance data was then log2 transformed in subsequent statistical analysis.

### Analyses of SILAC deep proteomics, mRNA stability half-life, and ribo-seq in *Mettl3* KO and WT mESCs

We reanalyzed our previously published data^8^. The unprocessed raw data of these datasets were previously deposited in NCBI GEO series GSE61998, and the processed data is available here as a source data file.

### Western blot analyses

Samples were separated on 10% (w/v) polyacrylamide Bis-Tris gels (Invitrogen) and transferred onto nitrocellulose membrane using iBlot gel transfer system (Invitrogen) set to P3 for 8 min with iBlot gel transfer stacks (Invitrogen). Membranes were blocked in 5% BSA, 0.05% Tween-20 in PBS for 1 h, and then incubated overnight at 4 °C with anti-FTO antibody (Millipore, 5-2H10; 1 µg/mL); anti-FABP5 antibody (R&D AF-1476; 0.5 µg/mL), anti-FABP2 antibody (BOSTER, PB9943; 0.5 µg/mL); anti-Mettl3 (Proteintech Group 15073-1-AP; 1:3000); anti-HSC-70 (Santa cruse SC-7298; 1:200), and a-TUBULIN (sigma T9026; 1:500). Proteins were visualized using the SuperSignal West Pico Luminol/Enhancer solution (Thermo scientific).

### Statistical analysis

All statistical analyses (unless stated otherwise) were performed using the SPSS version 18 software for Statistical Computing.

### Reporting summary

Further information on research design is available in the Nature Research Reporting Summary linked to this article.

## Supplementary information

Supplementary Information

Description of Additional Supplementary Files

Data S1

Data S2

Data S3

Data S4

Data S5

Data S6

Reporting Summary

## Data Availability

Data supporting the findings of this work are available within the paper and its Supplementary Information files and data. Source data are provided with this paper. A reporting summary for this article is available as a Supplementary Information file. All raw m^6^A/m-seq sequence reads data generated in this study have been deposited in NCBI SRA database under accession code PRJNA701370. All raw mass spectrometry proteomics data have been deposited to the ProteomeXchange Consortium via the PRIDE partner repository with dataset identifiers PXD029217, PXD029268, and PXD029280. The processed proteomics profiling data have been deposited in Zenodo database under accession code 10.5281/zenodo.5339101. RNA Stability and ribo-seq assays were previously deposited in^8^ via NCBI GEO series GSE61998. The processed source data underlying Figs. 1c, d, f–j, 2a–c, 3a–e, 4a–d, 5a, c–f; as well as Supplementary Figs. S1a−e, S2a, b, S3b, S4a−c, and S5b are provided as a Source data file. Source data are provided with this paper.

## Source data

Source data file
