# Supplementary material for: Dynamic regulation of N6,2′-O-dimethyladenosine (m6Am) in obesity
Source: Nat Commun. 2021 Dec 10;12:7185. doi: 10.1038/s41467-021-27421-2 (PMC8664860; doi:10.1038/s41467-021-27421-2)
Supplement: Supplementary file 1 — Supplementary Information [file 41467_2021_27421_MOESM1_ESM.pdf]

Supplementary Information File

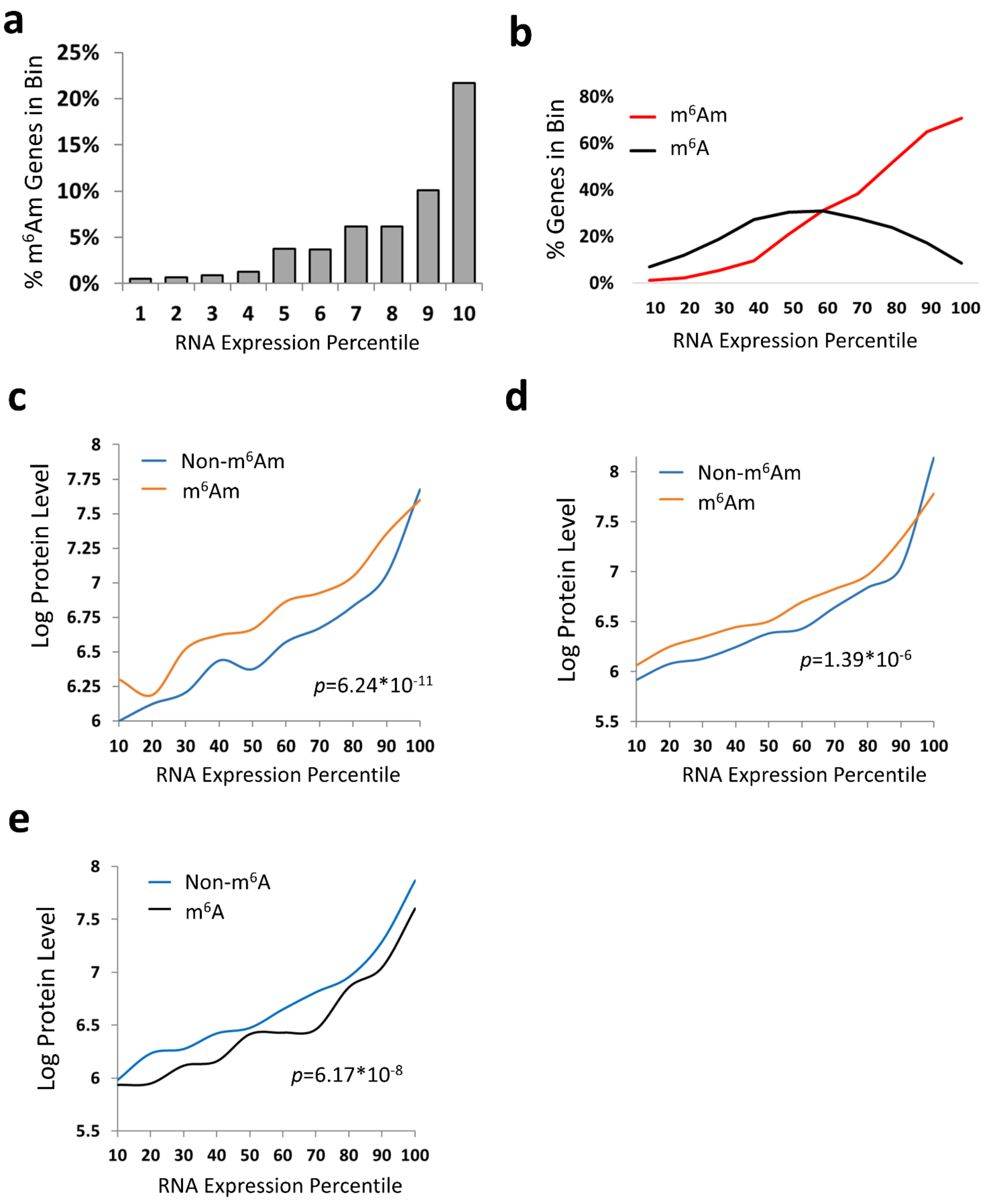

Supplementary Fig. 1 Protein and mRNA expression profiles of m^6Am- and non-5' UTR m^6A-modified genes in WT and *Mettl3* KO mESCs. a Fraction of m^6Am methylated genes across their RNA expression percentile bins after removing genes that were identified as m^6A decorated (sites located outside the 5' UTR) in WT mESCs. **b** Fraction of m^6Am and non- 5' UTR m^6A-methylated genes as a function of their RNA expression level in ES WT cells. **c** High-throughput protein expression of m^6Am- and non-m^6Am-methylated genes as a function of their RNA expression level in *Mettl3* KO mESCs**.** Two-tailed *p*-value of an ANCOVA analysis controlling for RNA levels of each gene as covariates is reported, $F(1,3765)=42.99, p=6.24X10^{-11}$. **d** High-throughput protein expressions of m^6Am- and non-m^6Am-methylated genes as a function of their RNA expression level in WT mESCs. Two-tailed *p*-value of an ANCOVA analysis controlling for RNA levels of each gene as covariates is reported, $F(1,3691)=23.36, p=1.39X10^{-6}$. **e** High-throughput protein expression of non- 5' UTR m^6A- and non-m^6A-methylated genes as a function of their RNA expression level in WT mESCs. Two-tailed *p*-value of an ANCOVA analysis controlling for RNA levels of each gene as covariates is reported, $F(1,3691)=29.43, p=6.17X10^{-8}$. Source data are provided as a Source Data file.

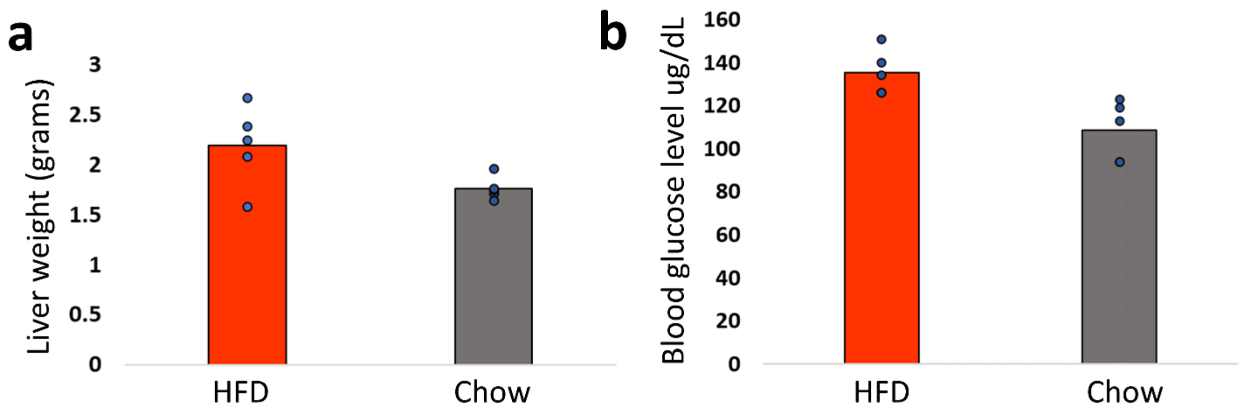

Supplementary Fig. 2 High fat diet mice physiologic parameters. **a** Liver weight of HFD mice and lean control chow diet mice. N=10 (5 HDF mice, and 5 Chow lean control mice). **b** Blood glucose levels of mice consuming HFD and control chow diet after 6-hour fasting. N=10 (5 HDF mice, and 5 Chow lean control mice). Source data are provided as a Source Data file.

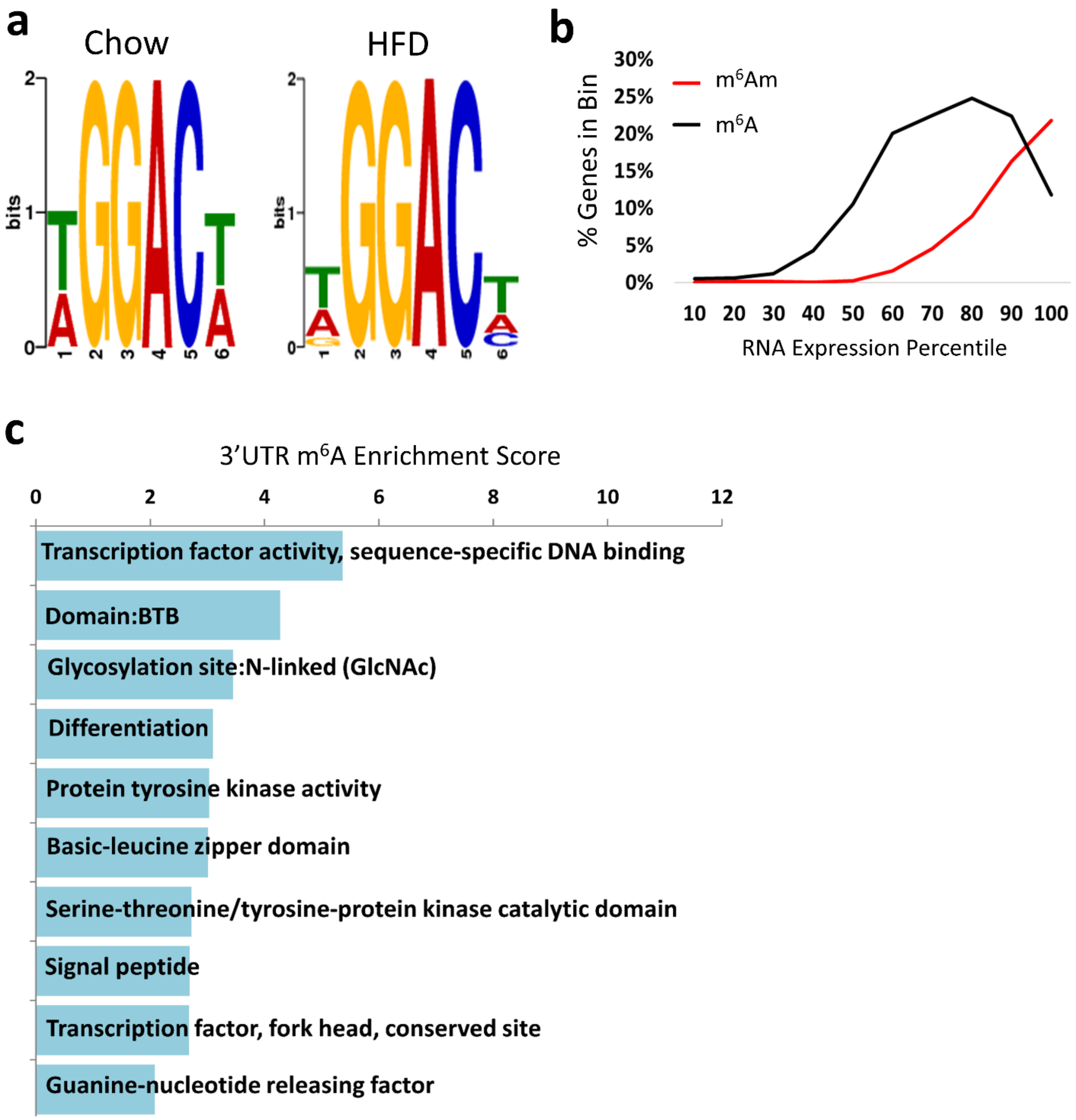

Supplementary Fig. 3 Extended profile of methylation characteristics of lean and HFD mice. a MEME RNA consensus motif of m^{6}A peaks in HFD and regular chow diet mice. **b** Fraction of m^{6}Am- and non- 5' UTR m^{6}A-methylated genes as a function of their RNA expression level in regular chow diet control mice. **c** Gene ontology analysis of m^{6}A 3' UTR showing an enrichment for non-metabolic processes. Source data are provided as a Source Data file.

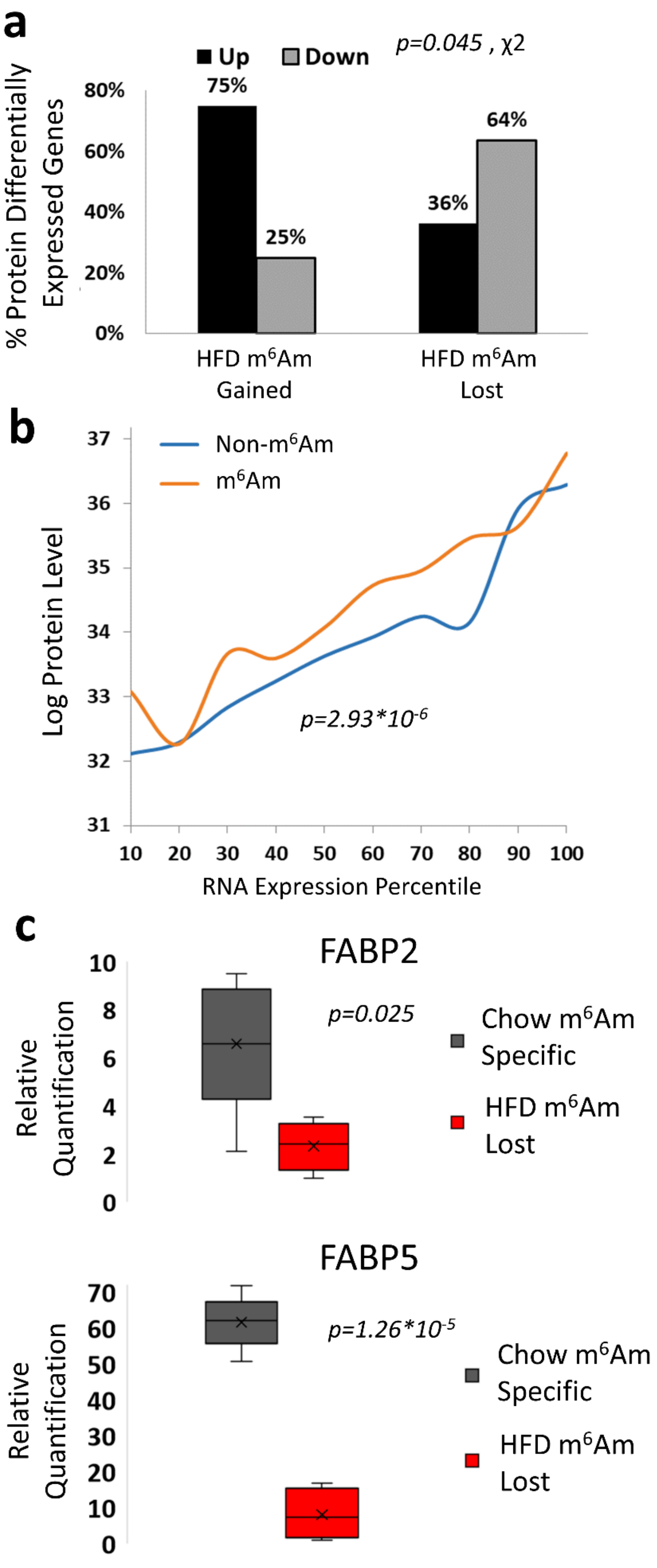
a
Up
Down
p=0.045 , χ2
% Protein Differentially Expressed Genes
80%
60%
40%
20%
0%
75%
25%
36%
64%
HFD m6Am Gained
HFD m6Am Lost
b
Non-m6Am
m6Am
Log Protein Level
37
36
35
34
33
32
31
p=2.93*10-6
10 20 30 40 50 60 70 80 90 100
RNA Expression Percentile
c
FABP2
Relative Quantification
10
8
6
4
2
0
p=0.025
Chow m6Am Specific
HFD m6Am Lost
FABP5
Relative Quantification
70
60
50
40
30
20
10
0
p=1.26*10-5
Chow m6Am Specific
HFD m6Am Lost

Supplementary Fig. 4 Extended protein regulation data of m^6Am genes in HFD mice. a High throughput proteomic profiling proportions of genes with protein differential expression (>1.5 fold) which gained or lost m^6Am upon HFD, after removing all >1.5 fold RNA differentially expressed genes. Two-tailed *p*-values are indicated (Chi-square test). **b** High-throughput protein expressions of m^6Am and non-m^6Am methylated genes as a function of their RNA expression level in regular chow diet control mice. Two-tailed *p*-value of an ANCOVA analysis controlling for RNA levels of each gene as covariates is reported, $F(1,2176)=21.98, p=2.93X10^{-6}$. **c** Quantification of FABBP2 and FABP5 western blots signal relative to the normalizing gene HSP-70. The results show a clear repeating pattern of overexpression across all lean biological replicates (N=5) versus all fat biological replicates (N=4). *p*-values are indicated (two tailed student t-test). Box plot surrounds the 1-3 quartiles, whiskers denote 1.5 interquartile range. Source data are provided as a Source Data file.

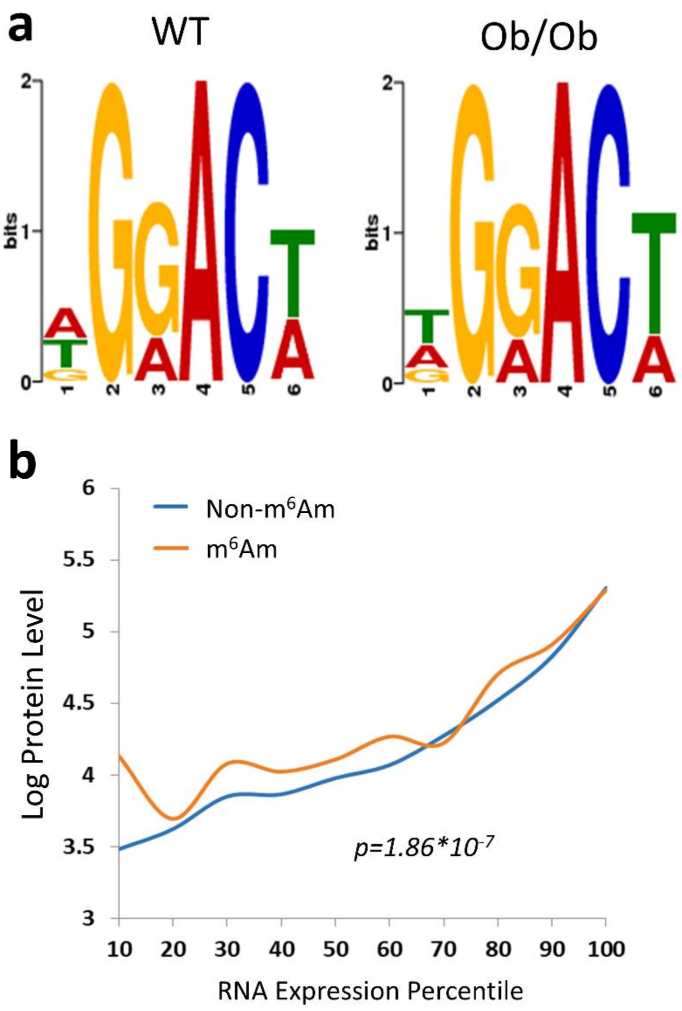

Supplementary Fig. 5 Extended characteristics of m^{6}Am genes in ob/ob and WT control mice. a MEME RNA consensus motif of m^{6}A peaks in ob/ob and their WT littermates mice. **b** High-throughput protein expressions of m^{6}Am and non-m^{6}Am methylated genes as a function of their RNA expression level in the WT control mice. Two-tailed *p*-value of an ANCOVA analysis controlling for RNA levels of each gene as covariates is reported. Source data are provided as a Source Data file.

Supplementary Table 1 Gene ontology enrichment terms identified for m^6Am genes in lean and HFD mice in this study (Fig. 2e) and their co-occurrence in *Pcif1* KO studies.

**Enriched m^6Am Process**	**Enriched in Reference**
Mitochondrion	22,23,24,25
Oxidation-reduction process	23,24,25
Endoplasmic reticulum	23,24,25
Fatty acid metabolism	23,24,25
Citrate cycle (TCA cycle)	23,24
Pyridoxal phosphate-dependent transferase	23,25
Peroxisome	23,25
Drug metabolism - cytochrome P450	23,24,25
Iron ion binding; Cytochrome P450; Monooxygenase activity; Linoleic acid metabolism	23,24,25
Non-Alcoholic Fatty Liver Disease (NAFLD); Alzheimer's disease; Respiratory chain	23,24,25
