# Supplementary material for: Dynamic regulation of N6,2′-O-dimethyladenosine (m6Am) in obesity
Source: Nat Commun. 2021 Dec 10;12:7185. doi: 10.1038/s41467-021-27421-2 (PMC8664860; doi:10.1038/s41467-021-27421-2)
Supplement: Supplementary file 2 — Description of Additional Supplementary Files [file 41467_2021_27421_MOESM2_ESM.docx]

**Description of Additional Supplementary Files**

**Title: Supplementary Data 1**

**Description:** m^6^Am MACS2 Peaks <50 nucleotides of a known TSS in *Mettl*3 KO mESCs. Excel spreadsheet.

**Title: Supplementary Data 2**

**Description:** Identified m^6^Am peaks in HFD and regular chow diet control mice. Excel spreadsheet.

**Title: Supplementary Data 3**

**Description:** Identified non-5’ UTR m^6^A MACS2 Peaks in HFD and regular chow diet control mice. Excel spreadsheet.

**Title: Supplementary Data 4**

**Description:** Gene ontology enrichment terms identified for m^6^Am genes in lean and HFD mice in this study (Fig. 2e) and their co-occurrence in *Pcif1* KO studies.

**Title: Supplementary Data 5**

**Description:** Identified MACS2 Peaks in HepG2 cells upon *FTO* overexpression, *FTO* knockdown, and control cells. Excel spreadsheet.

**Title: Supplementary Data 6:**

**Description:** Identified m^6^Am peaks in ob/ob and their WT littermates control mice. Excel spreadsheet.

**Title: Supplementary Data 7:**

**Description:** Identified m^6^A non- 5’ UTR MACS2 Peaks in ob/ob and their WT littermates control mice.
