# Supplementary material for: Dynamic regulation of N6,2′-O-dimethyladenosine (m6Am) in obesity
Source: Nat Commun. 2021 Dec 10;12:7185. doi: 10.1038/s41467-021-27421-2 (PMC8664860; doi:10.1038/s41467-021-27421-2)
Supplement: Supplementary file 9 — Reporting Summary [file 41467_2021_27421_MOESM9_ESM.pdf]

nature research

Corresponding author(s): Gideon Rechavi, Haim Cohen

Last updated by author(s): Sep 2, 2021

Reporting Summary

Nature Research wishes to improve the reproducibility of the work that we publish. This form provides structure for consistency and transparency in reporting. For further information on Nature Research policies, see our Editorial Policies and the Editorial Policy Checklist.

Statistics

For all statistical analyses, confirm that the following items are present in the figure legend, table legend, main text, or Methods section.

n/a	Confirmed	
☐	☒	The exact sample size (*n*) for each experimental group/condition, given as a discrete number and unit of measurement
☐	☐	A statement on whether measurements were taken from distinct samples or whether the same sample was measured repeatedly
☐	☒	The statistical test(s) used AND whether they are one- or two-sided<br>*Only common tests should be described solely by name; describe more complex techniques in the Methods section.*
☐	☒	A description of all covariates tested
☐	☒	A description of any assumptions or corrections, such as tests of normality and adjustment for multiple comparisons
☐	☒	A full description of the statistical parameters including central tendency (e.g. means) or other basic estimates (e.g. regression coefficient) AND variation (e.g. standard deviation) or associated estimates of uncertainty (e.g. confidence intervals)
☐	☒	For null hypothesis testing, the test statistic (e.g. *F*, *t*, *r*) with confidence intervals, effect sizes, degrees of freedom and *P* value noted<br>*Give P values as exact values whenever suitable.*
☐	☐	For Bayesian analysis, information on the choice of priors and Markov chain Monte Carlo settings
☐	☐	For hierarchical and complex designs, identification of the appropriate level for tests and full reporting of outcomes
☐	☒	Estimates of effect sizes (e.g. Cohen's *d*, Pearson's *r*), indicating how they were calculated

Our web collection on statistics for biologists contains articles on many of the points above.

Software and code

Policy information about availability of computer code

Data collection	Sequencing were collected using Illumina HiSeq 2500.
Data analysis	Statistical analyses were performed using the SPSS software for Statistical Computing version 18 and processed in Microsoft Excel. Sequences alignment were conducted using TopHat2. m6A peak enrichment analyses were conducted using MACS2 (version 2.1.0.20140616). RNA expression levels were calculated using CUFFLINKS (version 2.2.1) and differential expressions were done using its DESeq Bioconductor package. Gene ontology analyses were conducted using DAVID (http://david.abcc.ncifcrf.gov). Consensus motif searches were conducted using MEME (https://meme-suite.org/meme/). Protein and mass spectrometry analyses were conducted using Expressionist® software version 14 (Genedata, Switzerland) and Mascot v2.5.1 (Matrix Sciences). peptide search results were filtered using the PeptideProphet algorithm. ImageJ (version 1.46r) was used for western blot image analyses.

For manuscripts utilizing custom algorithms or software that are central to the research but not yet described in published literature, software must be made available to editors and reviewers. We strongly encourage code deposition in a community repository (e.g. GitHub). See the Nature Research guidelines for submitting code & software for further information.

Data

Policy information about availability of data

All manuscripts must include a data availability statement. This statement should provide the following information, where applicable:

- Accession codes, unique identifiers, or web links for publicly available datasets
- A list of figures that have associated raw data
- A description of any restrictions on data availability

Data supporting the findings of this work are available within the paper and its Supplementary Information files and tables. Source data are provided with this paper. A reporting summary for this article is available as a Supplementary Information file. All raw m6A/m-seq sequence reads data generated in this study have

been deposited in NCBI SRA database under accession code PRJNA701370 . All proteomic profiling data generated in this study have been deposited in Zenodo database under accession code 10.5281/zenodo.5339101. RNA Stability and ribo-seq assays were previously deposited in 8 via NCBI GEO series GSE61998. The processed source data underlying Figs 1c,d, f-j; 2a-c; 3a-e; 4a-d; 5a, c-f; as well as Supplementary figs S1a-e; S2a,b; S3b; S4a-c; and S5b are provided as a Source data file.

Field-specific reporting

Please select the one below that is the best fit for your research. If you are not sure, read the appropriate sections before making your selection.

- [x] Life sciences
- [ ] Behavioural & social sciences
- [ ] Ecological, evolutionary & environmental sciences

For a reference copy of the document with all sections, see nature.com/documents/nr-reporting-summary-flat.pdf

Life sciences study design

All studies must disclose on these points even when the disclosure is negative.

Sample size	Sample size was determined based on previously published studies and what is routinely used for the respective assay to ensure reproducibility of results (i.e., Dominissini et al, Nature 2012; Meyer et al.,Cell 2012; Geula et al., Science 2015). Typically 2-3 independent experimental samples were used in most assays.
Data exclusions	Peak calling analyses exceeded routinely used quality control cutoffs (i.e., FDR<5%, and minimum fold-change cutoffs) and are provided in the text next to the respective analysis.
Replication	None of the experiments reported had failed to replicate. Experiments were conducted in multiple biological replicates and several biological samples (for example m6A/m seq experiments were repeated in 3 HFD biological replicate mice and 3 lean control mice), and repeated also in another obesity mouse model (ob/ob) to ensure reproducibility.
Randomization	In our animal HFD experiment, mice were allocated to each group randomly. ob/ob mice were allocated based on their genetic profiling and were thus not randomized. In vitro molecular biological assays of cells were randomly assigned to control, overexpression, or knockdown conditions.
Blinding	Blinding was not possible without being exposed to the experimental treatments and outcomes, and thus the investigators were not blinded to sample allocation during experiments as well as outcome assessment. The results were obtained using objective quantitative methods.

Reporting for specific materials, systems and methods

We require information from authors about some types of materials, experimental systems and methods used in many studies. Here, indicate whether each material, system or method listed is relevant to your study. If you are not sure if a list item applies to your research, read the appropriate section before selecting a response.

Materials & experimental systems

n/a	Involved in the study	
[ ]	[x]	Antibodies
[ ]	[x]	Eukaryotic cell lines
[x]	[ ]	Palaeontology and archaeology
[ ]	[x]	Animals and other organisms
[x]	[ ]	Human research participants
[x]	[ ]	Clinical data
[x]	[ ]	Dual use research of concern

Methods

n/a	Involved in the study	
[x]	[ ]	ChIP-seq
[x]	[ ]	Flow cytometry
[x]	[ ]	MRI-based neuroimaging

Antibodies

Antibodies used	anti-m6A polyclonal antibody (Synaptic Systems, cat. no. 202 003);<br>anti-Fto antibody (Millipore, 5-2H10);<br>anti-Fabp5 antibody (R&D AF-1476);<br>anti-Fabp2 antibody (BOSTER, PB9943);<br>anti-Hsc-70 (Santa cruse SC-7298);<br>anti-a-Tubulin (sigma T9026);<br>anti-Mettl3 (Proteintech Group 15073-1-AP).
Validation	All antibodies used in this study are commercially available and were validated by the commercial suppliers:<br>anti-Fto antibody (Millipore, 5-2H10): Knockout validated for Western Bolting; reacts with Human, Rat, and Mouse (species of origin).<br>anti-Fabp5 antibody (R&D AF-1476): Knockout validated for Western Bolting; reacts with Human, Rat, and Mouse. In Western blots,

less than 5% cross-reactivity with recombinant mouse FABP4 and recombinant human (rh) FABP3 is observed and less than 1% cross-reactivity with recombinant rat (rr) FABP2 and rrFABP1 is observed.
anti-Fabp2 antibody (BOSTER, PB9943):Negative and Positive control validated for Western Bolting; reacts with Human, Rat, and Mouse. No cross reactivity with other proteins observed.
anti-Hsc-70 (Santa cruse SC-7298): Validated use in Western Bolting; reacts with Human, Rat, and Mouse; cited in 680 publications.
anti-a-Tubulin (sigma T9026): Validated use in Western Bolting; reacts with mouse and other species.
anti-Mettl3 (Proteintech Group 15073-1-AP): KD/KO Validated use in Western Bolting; reacts mouse and other species.

Eukaryotic cell lines

Policy information about cell lines

Cell line source(s)	HepG2 cell lines were originally obtained from ATCC. Eembryonic cell lines were derived from E3.5 C57BL/6 mouse embryos.
Authentication	none of the cell-lines used was authenticated
Mycoplasma contamination	Cells were routinely checked for mycoplasma contamination and were tested negative
Commonly misidentified lines (See ICLAC register)	No commonly misidentified cell lines were used in the study

Animals and other organisms

Policy information about studies involving animals; ARRIVE guidelines recommended for reporting animal research

Laboratory animals	6 Male C57BL/C mice (4 weeks); 3 ob/ob mice (13 weeks-old; 2 males; 1 female), as well as 3 WT litter mates (13 weeks-old; 2 males, 1 female) were used and sequenced in this study; an additional 3 ob/ob mice (1 male,13 weeks-old; and 2 females, 36 weeks-old) were used for an extended western blot.
Wild animals	We did not use wild animals in our study
Field-collected samples	We did not collect field samples in our study
Ethics oversight	All experiments were conducted in accordance with the Institutional Animal Care and Use Committee at Bar Ilan University

Note that full information on the approval of the study protocol must also be provided in the manuscript.
